# Developing a Gender Equity, Diversity, and Inclusivity (GEDI)-responsive curriculum framework for Philippine higher education: a qualitative case study of faculty perspectives

**DOI:** 10.3389/fsoc.2025.1672056

**Published:** 2026-01-07

**Authors:** Nilo Jayoma Castulo, Jayson Luciano De Vera, Ma. Laarni Buenaventura, Starr Clyde Lumanta Sebial, John Michael Del Rosario Aquino, Brenda O. Bua-ay, Rowena Raton Hibanada, Princess Zarla J. Raguindin, Zyralie Bedural, Raquel R. Geronimo, Lallen B. Quismundo, Emylin T. Batulat, Iona Ofelia Bathan Zanoria

**Affiliations:** 1 Department of Educational Leadership and Professional Services, College of Education, Mindanao State University – Tawi-Tawi College of Technology and Oceanography, Bongao, Philippines; 2 Faculty of Science, Technology and Mathematics, Philippine Normal University, Manila, Philippines; 3 College of Teacher Development, Philippine Normal University, Manila, Philippines; 4 School of Teacher Education, JH Cerilles State College, Zamboanga, Philippines; 5 College of Arts and Sciences, Laguna State Polytechnic University, Santa Cruz, Laguna, Philippines; 6 Philippine Normal UniversitySouth Luzon, Manila, Philippines; 7 College of Advanced Studies, Philippine Normal University, Manila, Philippines; 8 College of Teacher Development, Philippine Normal University, Manila, Philippines; 9 College of Teacher Development, Philippine Normal University, Manila, Philippines; 10 Philippine Normal University North Luzon, Manila, Philippines; 11 Philippine Normal University Mindanao, Manila, Philippines; 12 Philippine Normal University Visayas, Manila, Philippines; 13 Gender Equity, Diversity, and Inclusion Office, Philippine Normal University, Manila, Philippines

**Keywords:** gender equity, diversity, inclusivity, GEDI-responsive curriculum framework, higher education, curriculum framework, Philippines

## Abstract

**Introduction:**

Gender Equity, Diversity, and Inclusivity (GEDI) have become essential components of higher education reform; however, their integration into Philippine higher education curricula remains inconsistent. Thus, this study explored GEDI faculty members’ perspectives on integrating GEDI concepts into higher education. It proposes a responsive curriculum framework aligned with national mandates and global sustainable development goals.

**Methodology:**

A descriptive qualitative case study was conducted involving 19 faculty members from various higher education institutions in the Philippines. Data were gathered through online Key Informant Interviews (KIIs) and relevant document reviews. Thematic analysis using Atlas.ti 25 guided the coding and interpretation processes, complemented by member checking, reflexivity through the COREQ checklist, and triangulation to strengthen the analytical rigor.

**Results:**

The findings revealed that faculty members perceived GEDI integration as largely symbolic, with vague mentions in syllabi but insufficient curricular outcomes. Key gaps included (1) uneven implementation across disciplines (stronger in Social Sciences/Education vs. STEM), (2) non-standardized GEDI strategies, (3) faculty resistance and inadequate training, (4) marginalization of underrepresented identities in content, and (5) weak policy enforcement. A four-layer GEDI-Responsive Curriculum Framework (*macro, meso, micro, nano*) was proposed to embed intersectionality, contextual relevance, and accountability across all educational levels. This research bridges policy-practice gaps by aligning with the local and international higher education curriculum and emphasizing intersectionality, localized reforms, and measurable competencies (e.g., empathy, critical gender consciousness). The findings of the study are context-specific to selected Philippine regions, and broader applicability requires further validation. Underrepresented contexts (e.g., Indigenous Peoples and disability-specific programs) were minimally covered. Future research should broaden geographic coverage and pilot systematic feedback systems to evaluate the applicability and sustainability of the framework across diverse higher education contexts.

## Introduction

The principles of Gender Equity, Diversity, and Inclusivity (GEDI) are vital for establishing educational settings that are inclusive, equitable, and supportive, ensuring that all students have equal opportunities regardless of their gender, race, or socioeconomic status (Benslimane and Moustaghfir, 2020; Page and Takkenberg, 2025; Raton-Hibanada et al., 2025). In higher education, the GEDI principles aim to cultivate an academic atmosphere that is fair, diverse, and inclusive. These principles are crucial for promoting social empowerment and moving towards a more inclusive society (Page and Takkenberg, 2025). Integrating gender equity throughout the curriculum is necessary to tackle systemic biases and encourage inclusive teaching methods (Peña et al., 2021; Samuel et al., 2025). For instance, adopting teaching strategies that cater to diverse learning needs and offer additional support to marginalized students is one approach (Kim et al., 2023).

Importantly, the integration of the GEDI offers crucial support to underrepresented communities. A comprehensive and high-quality education that addresses gender identity and sexual orientation is vital for achieving equity and equality among lesbian, gay, bisexual, transgender, intersex, query or questioning, and plus (LGBTIQ+) students. Curricula that thoroughly address these issues positively impact both LGBTIQ+ and non-LGBTIQ+ students, fostering a genuinely inclusive educational environment (Palacios-Hidalgo and Huertas-Abril, 2023). Higher education continues to struggle with persistent systemic and structural barriers deeply rooted in its historical framework, which has historically neglected diversity. This has led to ongoing inequities even in the aftermath of civil rights advancements (Copeland and Tarver, 2020). For example, entrenched patriarchal norms further hinder gender inclusivity, underscoring the need to integrate equity comprehensively across the curriculum, rather than relying on standalone courses (Samuel et al., 2025). The lack of integrated gender awareness poses a significant challenge; although stakeholders may show sensitivity, the curriculum often fails to adequately incorporate it or to feature representative voices and diverse faculties (Samuel et al., 2025).

Higher education institutions (HEIs) encounter a variety of challenges when attempting to integrate Gender Equity, Diversity, and Inclusivity (GEDI) into their systems. These challenges are complex and arise from systemic, cultural, and institutional barriers. Many institutions face challenges owing to limited resources, hindering the implementation of comprehensive GEDI programs. This includes financial constraints and inadequate support services (Roy, 2025). In addition, the rigidity of the current curricula often obstructs the inclusion of GEDI principles, making it challenging to incorporate these values into educational content and teaching methods (Roy, 2025; Samuel et al., 2025). Beyond the curriculum, there is a significant lack of representation of women and minorities in leadership roles in HEIs. This absence of diversity in leadership positions sustains existing inequalities and diminishes the impact of GEDI efforts (Gustafson and Lee-Johnson, 2023; Harper et al., 2021). Additionally, students and faculty who experience intersectional forms of exclusion (e.g., based on gender, race, and ethnicity) often face compounded challenges and discrimination, which are not sufficiently addressed by current GEDI initiatives (Aksay Aksezer et al., 2023; Jean-Marie, 2011). Other contributing factors include cultural and societal influences, a lack of understanding of the GEDI data, persistent gender inequalities, the intersection of various vulnerabilities, limitations in data collection and analysis, hidden power dynamics, insufficient funding and recognition, and challenges in curriculum integration (Waldman et al., 2023). Therefore, this study explored GEDI faculty members’ perspectives on integrating GEDI concepts into higher education. This study seeks to answer the following research question:

1. What are faculty members’ perceptions of the integration of GEDI concepts within the higher education curriculum framework?
2. What are the identified gaps and challenges in integrating the GEDI principles within existing curricular structures?

## Literature review

### Perceptions of GEDI integration in higher education curriculum

A study conducted with faculty members in the Philippines revealed that they intentionally incorporated Gender and Development (GAD) principles by tailoring course content, engaging in reflective activities, and facilitating discussions that challenge stereotypes and promote empathy. They also demonstrate gender-responsive behavior by employing inclusive language and ensuring equitable interactions in the classroom (Legarde et al., 2025). Despite significant progress, faculty members report that limited resources and training pose major obstacles to deeper integration of gender and cultural frameworks (Rodiguez et al., 2025). In addition, entrenched patriarchal norms and systemic barriers continue to impede gender inclusivity. Addressing these challenges requires more than just creating specific courses; it also necessitates a comprehensive, inclusive curriculum that embeds gender equity throughout higher education (Samuel et al., 2025). Insights from faculty and staff highlight ongoing issues related to gender equity. Faculty members note persistent gender disparities, with female faculty encountering more significant challenges and expressing greater concern than their male counterparts (Bordieri et al., 2024). Furthermore, educators in health professions have identified significant gaps, emphasizing the need for enhanced understanding and training in Diversity, Equity, and Inclusion (DEI) alongside cultural competence within their current curriculum and professional development (Ellis et al., 2023). Institutional and administrative perspectives refer to essential methodological changes. There is a strong argument for moving beyond isolated courses or initiatives to the thorough integration of gender equity principles across all facets of higher education (Samuel et al., 2025). Effective strategies for achieving this involve participatory approaches, as demonstrated by initiatives in Spain and Italy that successfully engage diverse stakeholders in developing gender-responsive curricula (Campanini Vilhena et al., 2024). The foundation of these initiatives is the recognized need for structural transformation. This is exemplified by frameworks developed in Canada for STEM education, which specifically aim to reform institutional structures to eradicate systemic discriminatory practices (Ruel and Tajmel, 2024).

### Identified gaps and challenges of integration of GEDI principles

Substantial challenges impede the successful incorporation of Gender Equity, Diversity, and Inclusivity (GEDI) principles across various sectors. Fundamental systemic obstacles sustain inequality, encompassing entrenched implicit biases such as systemic racism, sexism, and homophobia within organizational cultures. The presence of these biases adversely affects performance evaluations, promotion opportunities, and overall career advancement of marginalized groups (Miller and Mustapha, 2022). This form of discrimination permeates the healthcare sector, where transgender women face significant exclusion as a result of medical genderism (Shabalala and Campbell, 2023). Moreover, enduring cultural norms and gender stereotypes perpetuate bias, limiting personal opportunities and involvement in various domains such as athletics and professional settings (Akinniyi and Gang, 2024). Insufficient data gathering and portrayals hinder their advancement. The execution of robust GEDI policies is hindered by a lack of comprehensive data, which does not adequately reflect the existing disparities and biases. The absence of dependable information complicates the creation of targeted interventions and assessment of their effectiveness (Tiwana et al., 2024). This ongoing challenge is exacerbated by the persistent lack of representation of women and racial minorities in leadership roles and higher-paying careers. This lack continues to sustain disparities and undermines the efficacy of diversity efforts (Galbally et al., 2023; Tiwana et al., 2024). Numerous obstacles exist in the execution of Gender Equity, Diversity, and Inclusivity (GEDI) within higher education institutions in Southeast Asia (Waldman et al., 2023). Conventional gender roles and stereotypes persist in restricting women’s access to leadership positions, and insufficient analysis and interpretation of current GEDI data further intensifies these challenges (Waldman et al., 2023). Deficiencies in leadership and organizational structures create further challenges. To achieve true inclusivity, it is essential for leaders to acknowledge their biases and commit to significant organizational transformation. Nonetheless, only a limited number of leaders effectively implement these principles to enhance collaboration and create genuinely inclusive environments (Hellman, 2023). Numerous organizations often succumb to the pitfalls of tokenism, enacting surface-level GEDI policies that do not address the fundamental issues of inequality. This surface-level strategy may exacerbate feelings of exclusion among diverse individuals, instead of fostering genuine inclusion (Srinidhi, 2023; Tiwana et al., 2024).

## Methodology

### Gender Equity, Diversity, and Inclusivity (GEDI) context in the Philippines

In the Philippines, Gender Equity, Diversity, and Inclusivity (GEDI) are strongly reflected across SDG 5, 4, 10, 16, and 17, which together shape the Philippines’ overall performance in Sustainable Development Report (2025). The Sustainable Development Dashboard shows that SDG 5 (Gender Equality) in the Philippines is classified as *Challenges Remain*, with a *Moderately Improving* trend, indicating that while gender-related disparities and protection gaps persist, the country is steadily progressing due to ongoing reforms and strengthened gender policies. Similarly, SDG 4 (Quality Education) is rated as *Challenges Remain* and *Moderately Improving*, reflecting gradual gains in inclusive and equitable education, but also highlighting continuing concerns in learning outcomes, gender-responsive education, and access for marginalized groups. By contrast, SDG 10 (Reduced Inequalities) faces *Major Challenges* with a *Moderately Improving* trend, suggesting persistent structural inequalities affecting gender, income, ethnicity, and regional accessibility, with limited progress toward reducing systemic exclusion. SDG 16 (Peace, Justice, and Strong Institutions) is likewise categorized under *Major Challenges Remain*, paired with a *Stagnating* overall trend, underscoring ongoing issues in governance, institutional safety, justice mechanisms, and inclusive public systems, which directly impact the implementation of the GEDI in higher education. Meanwhile, SDG 17 (Partnerships for the Goals) falls under *Significant Challenges Remain* but shows a *Moderately Improving* trajectory, demonstrating that while the Philippines has strengthened coordination among government, academia, civil society, and international bodies, more robust and sustained multisector partnerships are required to fully advance Gender Equity, Diversity, and Inclusivity (GEDI) nationwide. Table 1 presents all relevant Philippine laws and the Commission on Higher Education (CHED) policies on Gender Equity, Diversity, and Inclusivity (GEDI) in Philippine higher education.

**Table 1:** Relevant Philippine laws and Commission on Higher Education Policies on Gender Equity, Diversity, and Inclusivity (GEDI) in Philippine higher education.

Year	Title of the policy	Key insights
1992	Republic Act 7192—Women in Development and Nation Building Act	The law requires government bodies to eliminate gender bias in their policies and initiatives, ensuring that women have equal opportunities to participate in development. This foundational legislation set the stage for integrating gender considerations across all sectors, including education, by advocating for equal chances for women.
1992	Republic Act 7277—Magna Carta for Disabled Persons	Forbids any educational institution from refusing admission to an individual due to a disability and mandates that schools address the unique needs of students with disabilities. Additionally, it instructs state universities and colleges to combat discrimination by creating supportive resources and inclusive initiatives for students with disabilities, thereby promoting inclusivity in higher education.
1995	Republic Act 7877—Anti-Sexual Harassment Act of 1995	In an educational context, sexual harassment is characterized by an authority figure, such as a professor or school official, requesting sexual favors in return for grades, scholarships, or other advantages. Schools are mandated to establish a Committee on Decorum and Investigation (CODI) to address complaints, thereby creating a system to safeguard students and staff from harassment based on gender.
2009	Republic Act 9710—Magna Carta of Women	A comprehensive law on women’s rights requires all government bodies, including CHED and state universities, to implement gender mainstreaming and gender-responsive initiatives. The law’s implementing rules specifically assign CHED the responsibility of creating gender-sensitive curricula, gender-equitable teaching resources, and gender awareness programs in higher education, thus embedding gender equity policies within universities.
2015	CHED Memorandum Order No. 1, s.2015—Policies and Guidelines on Gender and Development in CHED and HEIs	The initial directive from CHED regarding GAD set forth guidelines aimed at advancing gender equality and empowering women within higher education. It established Gender and Development (GAD) as a fundamental aspect within both the Commission and all Higher Education Institutions, mandating the inclusion of gender-responsive curricula, research, and extension programs, as well as the establishment of GAD Focal Point Systems on campuses. This policy effectively implemented the Magna Carta of Women within the higher education sector.
2016	Republic Act 10908—Integrated History Act of 2016	The law mandates the inclusion of the history, culture, and identity of Filipino-Muslims and Indigenous Peoples in the Philippine history curriculum at both the basic and higher education levels. This legislation aims to enhance cultural diversity and inclusion in universities by incorporating the viewpoints of marginalized cultural communities into educational materials, thereby promoting a deeper understanding and respect for diversity.
2019	Republic Act 11313—Safe Spaces Act (Bawal Bastos Law)	The scope of anti-sexual harassment policies is broadened to include not only superiors but also colleagues, addressing gender-based harassment in educational settings, workplaces, public spaces, and online platforms. In schools, it requires the implementation of more stringent anti-harassment measures, such as a code of conduct, awareness initiatives, and the incorporation of gender sensitivity into the curriculum. This legislation seeks to create safe and inclusive campuses by penalizing all types of sexual harassment, including sexist, homophobic, or transphobic behavior, and empowering victims to pursue justice.
2022	CHED Memorandum Order No. 3, s.2022—Guidelines on Gender-Based Sexual Harassment in HEIs	This directive, aligned with the Safe Spaces Act, outlines comprehensive instructions for higher education institutions to both prevent and address gender-based sexual harassment. It mandates that all HEIs implement policies and systems, such as enhanced CODI procedures, to penalize sexual harassment on campus, protect the rights of students and staff, and foster a secure, gender-sensitive academic setting.
2025	PCW-DepEd-CHED-TESDA Joint Memorandum Circular 2025-03—Gender-Responsive Assessment Tools	A directive involving multiple agencies has established a cohesive framework for assessing textbooks and educational materials to ensure they are devoid of gender bias and stereotypes. This policy requires the implementation of gender-responsive evaluation tools across basic, higher, and technical-vocational education, thereby fostering diversity and inclusion within the curriculum. It aims to nurture gender-equitable and inclusive viewpoints among students.

Source: CHED Memo No. 1 (2015); CHED Memo No. 3 (2022); Joint Memorandum Circular No. 3 (2025); Republic Act No. 7192 (1992); Republic Act No. 7277 (1992); Republic Act No. 7877 (1995); Republic Act No. 9710 (2009); Republic Act No. 10908 (2016); Republic Act No. 11313 (2019).

#### Research design

This study employed a descriptive qualitative case approach. The qualitative case study methodology (QCSM) is a research approach designed to explore complex phenomena within real-life contexts using multiple data sources and collection methods (Merriam and Tisdell, 2016; Yin, 2018). Using a case study design, we employed multiple methods, including Key Informant Interviews (KIIs) and collection of additional documents relevant to the research. To ensure the rigor and trustworthiness of the findings generated through this methodology, researchers have employed specific validation strategies. These include reflexivity the Consolidated Criteria for Reporting Qualitative Research (COREQ) checklist, member checking (verifying interpretations with participants), and triangulation (using multiple data sources, such as policy documents). These techniques collectively enhance the credibility and reliability of a study’s outcomes (Crowe et al., 2011; Denzin and Lincoln, 2018).

#### Participants and sampling

**Figure 1:**
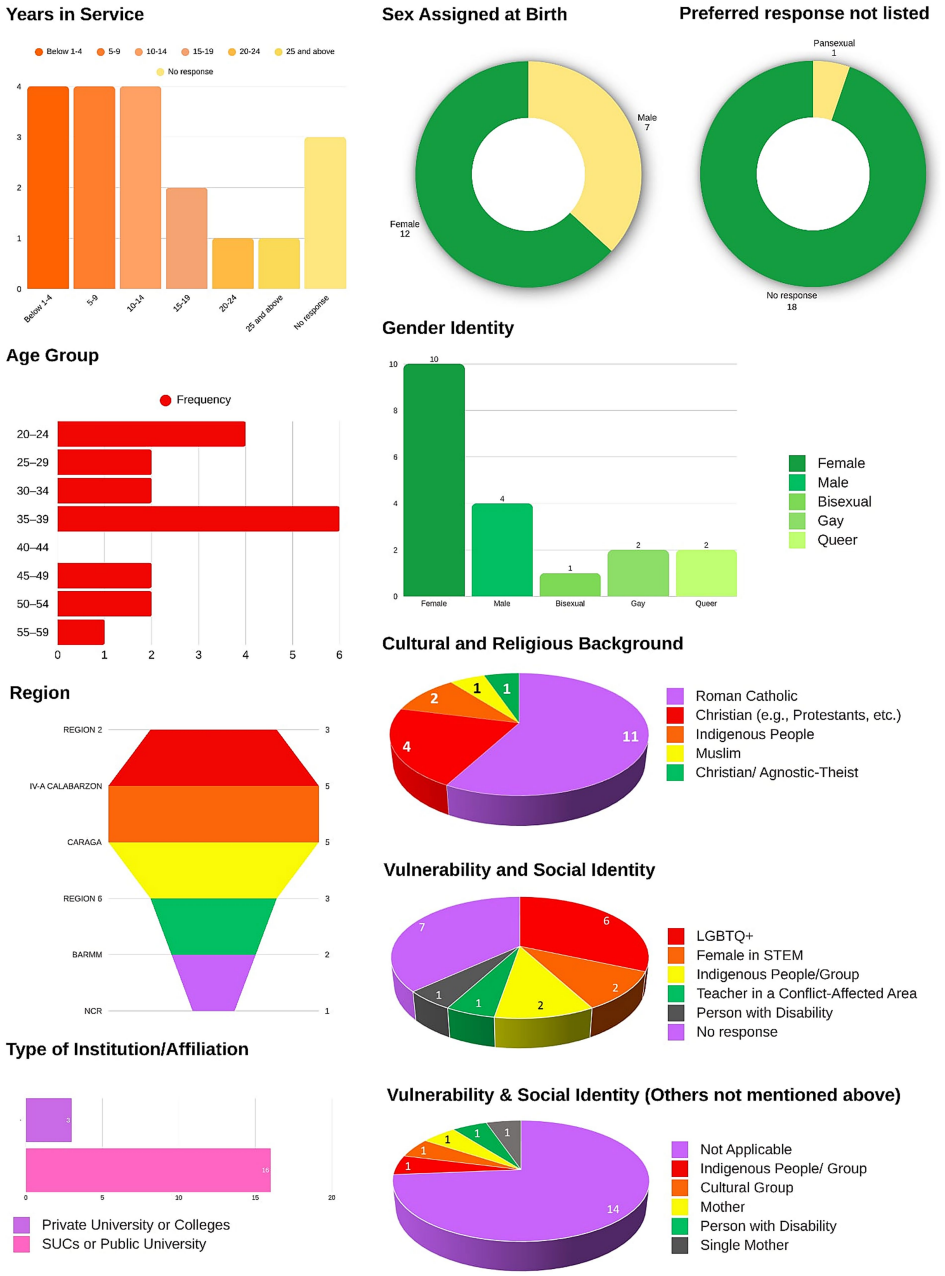
Demographic profiling of the participants. Authors’ own work.

Figure 1 shows the demographic profiles of diverse groups of participants. We ensured a purposive sampling technique that focuses on faculty members with a background in the curriculum at higher education institutions. These faculty members hold various designations at their respective universities. Most participants were early career professionals, with the largest groups having below 1–4 and 5–9 years of service. A few have between 10 and 14 years, 15 and 19 years, and 25 years and above, indicating that participants are primarily young educators, but with a small mix of mid-career and senior practitioners. The majority of participants were female (12) compared to males (7). This aligns with the typical gender distribution in education-related fields, which are often female-dominated. A significant proportion (18 participants) did not specify an “other” preferred response, while a very small percentage selected pansexual. This finding suggests that most participants fit within the provided categories and did not feel the need to choose an alternative identity descriptor. The participants were largely young adults, with the largest cluster in the 35–39 age range, followed by those aged 20–24 and 25–34 years. Few participants fell into the older age category (40 and above). This further supports the fact that the sample was composed mostly of younger or early career educators. Most participants were female (*n* = 10), followed by males (*n* = 4). There are also individuals identified as bisexual, gay, and queer, reflecting the presence of gender-diverse and LGBTQ+ identities within the group. Participants came from various regions across the Philippines, with the highest numbers from Region IV-A (CALABARZON) and CARAGA, followed by Region 2 and Region 6. There is representation from BARMM, NCR, and Region 2, showing wide geographic coverage and participation. Most participants are employed in SUCs or public universities (16), and a smaller number come from private universities or colleges (3). This indicates that the study largely reflects perspectives of the public higher education sector. The participants predominantly identified as Roman Catholic (11), which was expected given the Philippine religious landscape. Other representations include Christian denominations, indigenous peoples, Muslims, and Christian/Agnostic-Atheists, showing a diversity of faith and cultural backgrounds. This study focused specifically on faculty and HEI staff perspectives regarding GEDI integration and did not include student interview data. References to student experiences reflect faculty observations rather than primary student testimonies.

#### Data collection procedures

Prior to gathering data, the researchers secured approval from the Research Ethics Committee at the Philippine Normal University (REC Code No. 2025-106) to carry out the study. The committee assessed both the proposal’s content and ethical aspects of the research. Once the project was completed, the researchers submitted the final research to the ethics committee for endorsement. Consequently, this study forms part of a larger research project sponsored by the Southeast Asian Ministers of Education Organization (SEAMEO) Regional Center, specializing in higher education and development (RIHED). After receiving clearance, we conducted online interviews via the Zoom platform using Key Informant Interviews (KIIs). Given the participants’ professional roles, Zoom facilitated remote participation, while preserving the depth of inquiry during the interviews. Each interview lasted between 40 min and 1 h. The researchers ensured that all the participants were university-level curriculum experts with direct knowledge or experience pertinent to the research. Each session was manually transcribed for analysis, with everyone is consent to record. This approach ensured the collection of comprehensive and qualitative data essential for meeting the objectives of the study. Thematic analysis was performed using Atlas.ti version 25. Following the initial coding process, as per the guidelines of Clarke and Braun (2017), the researcher engaged in member checking with some participants based on the generated codes and finalized the themes through a series of discussions. The researchers also reviewed the consolidated criteria for Reporting Qualitative research (COREQ) checklist as part of their reflexivity process.

## Results

### What are the faculty members’ perceptions of the integration of the GEDI concepts within the higher education curriculum framework?

#### Visibility and language of GEDI in the university curriculum

Faculty members perceive the integration of GEDI concepts as a critical step towards creating a more inclusive educational environment. They highlight that the visibility of these themes in the curriculum is not merely about compliance, but about genuinely embedding these values into the educational framework. The language used in the teaching materials plays a significant role in shaping students’ understanding and acceptance of diversity. By using inclusive languages and representing various identities fairly, educators can challenge stereotypes and promote a culture of respect and equality. Participants revealed that GEDI-related terms are often present in curriculum documents or course syllabi but are articulated in vague or symbolic ways. These mentions are typically embedded in institutional missions or graduate attribute statements, without corresponding course-level outcomes or assessment strategies. Moreover, Gender equity, when present, is often framed as a value under ethics or civics education rather than as a competency with explicit learning outcomes. This leads to a situation where gender-related topics are not consistently evaluated or taught across subjects. This creates a gap between declared values and actual instructional practices.

*“We aim to integrate gender responsiveness through language by ensuring that there is neutrality and sensitivity, and that there is equal representation of various genders. Gender in language must be expressed with equal value and dignity,”*

*“Content should specifically focus on diversity, equality, and inclusion, with due respect to intersectionality. It also includes the portrayal of images, illustrations, and even color choices, making sure that all elements are appropriately portrayed, made visible, and well-represented,”*

*“We talk about respect, diversity, and tolerance in values education, but these are not directly measured. Gender is part of that, but never separately addressed,”*

#### Holistic support systems

Participants recognized that a holistic approach to support systems is essential for the successful integration of GEDI principles in higher education. This involves creating an environment in which students feel valued and supported, which goes beyond mere access to educational resources. It includes emotional, social, and academic support tailored to students’ diverse needs. Faculty members advocate policies and practices that address the unique challenges faced by various student demographics, including those from marginalized communities, and emphasize the importance of ongoing training and awareness for educators to effectively implement these support systems. For instance, while financial assistance and scholarships are crucial, they often do not translate into long-term employment or sustainable livelihoods without the accompanying support structures that foster skill development and integration into the workforce.

*"The core issue here is support—not just access to education, but support systems that enable long-term success. We must empower students beyond just providing them with resources; we need to ensure they have the emotional and social support to thrive."*

*"Even with policies like UNIFAST and free higher education in place, we continue to uphold equity in our admission processes. This means giving priority to marginalized sectors in society—ensuring they are not left behind, but are given meaningful opportunities to access higher education."*

*"There should be regular training and workshops about SOGIESC. A safe space and support networks for everyone to openly discuss GEDI issues. This support will empower teachers to create classrooms that are safe, inclusive, and affirming for all learners."*

#### Inclusive curriculum models

This theme reflects faculty members’ perceptions regarding the integration of Gender Equity, Diversity, and Inclusivity (GEDI) concepts within the higher education curriculum framework. Faculty members emphasize the importance of developing curriculum models that are not only inclusive but also responsive to the diverse needs of students. This involves embedding GEDI principles into all aspects of the curriculum to ensure that all students feel represented and valued. Participants viewed inclusive curriculum models as essential for fostering an educational environment that respects and promotes diversity. They believe that integrating GEDI concepts into the curriculum should not be an afterthought, but a foundational aspect of curriculum design. This includes not only the content taught but also the pedagogical approaches used to deliver that content. Faculty members advocate for a curriculum that reflects the realities of all students, including those from marginalized backgrounds, and actively dismantles barriers to learning. Many stakeholders have suggested a modular approach in which GEDI content is embedded into general education and core professional courses rather than being isolated in electives or standalone gender courses. This ensured that all students were exposed to gender-inclusive content, regardless of their major. Another curricular strategy was the design of interdisciplinary courses or capstone projects that frame GEDI issues within real-world contexts. The faculty highlighted that GEDI concerns are not discipline-specific, and thus lend themselves well to cross-cutting approaches that combine, for example, health, education, law, and technology.

*"The integration of gender perspectives is not just a suggestion—it is an institutional policy. Our office is part of the committee that reviews syllabi and instructional materials to ensure that they reflect gender perspectives.”*

*"We do not treat gender as a standalone topic, separate from other subjects. In fact, our university strongly believes that even subjects like mathematics and science can—and should—integrate gender perspectives.”*

### Intersectional and context-based curriculum reforms

The participants noted that reforms are needed to make culturally responsive practices more responsive to local contexts and lived experiences. Participants argued that a one-size-fits-all approach to education is inadequate; instead, curricula should reflect the complexities of students’ identities and experiences. This includes integrating themes of gender, race, class, and other social categories into the curriculum, ensuring that all students see themselves as represented and valued in their education. Faculty members advocate for ongoing training and resources to help educators effectively implement these reforms. Localized curriculum content allows students to engage with gender and diversity issues in ways that are relevant, concrete, and transformative. There was a strong call to include the perspectives and lived experiences of marginalized groups, such as LGBTQIA+, indigenous peoples, persons with disabilities, and students from disadvantaged socioeconomic backgrounds in the curriculum design and review processes.

*“If we contextualize GEDI to our community realities, students will see that these are not abstract issues but part of their lived experiences.”*

*“We need to hear from the communities most affected by exclusion to shape our curriculum.”*

*“We need GEDI integration not just in education or nursing, but in agriculture, engineering, and business. And these must be tied to local realities.”*

#### GEDI-aligned competencies and learning outcomes

Alignment is crucial to fostering an inclusive educational environment in which all students can thrive. This identifies the core outcome that a GEDI-responsive curriculum should foster empathy, critical thinking, and inclusive civic identity. This emphasizes that these competencies should be intentionally embedded in course outcomes to ensure alignment with the broader goals of inclusive education. The learning outcomes for students should reflect these competencies, ensuring that graduates are not only knowledgeable about GEDI issues, but also capable of applying this understanding in real-world contexts. Moreover, the successful integration of GEDI-aligned competencies into the curriculum is vital for preparing students to engage with and positively contribute to a diverse and equitable society. Faculty members consistently emphasized the need for learning outcomes that go beyond cognitive mastery and include socio-emotional and ethical competencies. Participants advocated embedding values, such as empathy, inclusivity, respect for diversity, and critical gender consciousness, in program outcomes.

*“Gender awareness should be a graduate attribute—not just knowledge of facts, but the capacity to analyze gender dynamics in our field.”*

*“The integration of gender perspectives is not just a suggestion—it is an institutional policy. Our office is part of the committee that reviews syllabi and instructional materials to ensure that they reflect gender perspectives.”*

### Monitoring, evaluation, and accountability mechanisms

Faculty members advocate the development of standardized tools and processes that can provide clear metrics for evaluating the inclusivity and responsiveness of educational materials and practices. This focus on accountability is viewed as a way to ensure that institutions remain committed to their GEDI goals and can make the necessary adjustments based on evidence and feedback. Effective monitoring and evaluation are essential to ensure that the integration of GEDI principles into curricula and teaching practices is not only implemented but also sustained over time. Stakeholders have proposed regular GEDI audits, including curriculum mapping exercises, syllabus reviews, and institutional self-assessment tools that incorporate feedback from students on their classroom experiences related to gender equity and inclusion. Collaboration with gender advocacy groups, NGOs, and interdisciplinary experts has been suggested as a mechanism for the independent and credible evaluation of GEDI efforts.

*"To address diversity, equity, and inclusion in teaching practices and curriculum development, there must be a tool to measure. If this is responsive, this is inclusive, and this is neutral. Because if we don't have that tool, of course, we may have different interpretations of this matter."*

*“Let’s not keep this internal. Partnering with experts ensures we don’t miss blind spots.”*

#### What are the identified gaps and challenges in integrating GEDI principles within existing curricular structures?

##### Uneven implementation across disciplines

According to the participants, the GEDI is more visible in Social Sciences and Teacher Education; it remains peripheral or implicit in Science, Technology, Engineering, and Mathematics (STEM) and technical disciplines, suggesting that implementation is discipline-dependent rather than systemic. This highlights significant disparities in how GEDI principles are integrated into curricula across various academic fields. This unevenness often stems from a lack of clear institutional policies and varying levels of commitment among faculty members, which can lead to the inconsistent application of GEDI concepts in different disciplines. For instance, while some departments may actively incorporate gender-responsive languages and diverse perspectives into their syllabi, others may neglect these elements, resulting in a curriculum that fails to represent the full spectrum of student identities and experiences. Participants consistently indicated that GEDI themes are more actively and structurally integrated in fields such as Social Sciences and Teacher Education, where the discussion of identities, culture, power, and diversity is inherent in the content. Conversely, STEM and technical programs exhibited minimal GEDI integration. Most participants indicated that these fields prioritized technical competencies, leaving little perceived room for social issues, such as gender and diversity.

*“In Education and Psychology, we talk about gender identity, inclusion, and SOGIESC. It’s part of our training and classes,”* one faculty member noted.

*“As an engineering faculty, I focus on outcomes like design and computation. GEDI isn’t really part of our course outcomes or activities,”* the STEM instructor commented.

##### Non-standardized GEDI strategies

This theme argues that integration often depends on individual faculty initiatives rather than institutional mandates, leading to uneven practices and a lack of cohesive strategy for embedding gender equity and inclusion within the curriculum. Implementation of the GEDI initiatives across educational institutions. The lack of a unified approach often results in varied interpretations and applications of the GEDI principles, leading to fragmented educational experiences for students. The absence of standardized policies and frameworks for GEDI implementation can lead to a lack of accountability and sustainability in these initiatives, making it difficult to measure their effectiveness or impact. Several participants noted that the inclusion of GEDI themes in the curriculum largely depended on faculty interest, awareness, or advocacy. There are cases in which educators design their own modules or incorporate gender perspectives through individual efforts. Participants emphasized that institutions rarely mandate GEDI as a curricular priority across all departments. There is often no clear policy or framework that guides integration, nor are accountability mechanisms in place to ensure its implementation.

*“I took it upon myself to add a unit on gender awareness in my Sociology class. But that’s not required—it’s my own initiative,” shared a professor.*

*“Our university has a GAD office, but they are more focused on events. There's no push to embed gender in the curriculum itself,” said one academic program head.*

##### Structural and institutional constraints

Faculty members noted that systemic barriers limit GEDI integration into curriculum planning. These include already congested curricula, institutional inertia, and the perception that the GEDI is non-essential, which collectively diminishes the prioritization of inclusive educational practices. These constraints often manifest as vague policies and lack of clear guidelines, leading to inconsistent interpretations and implementation of GEDI initiatives across institutions. For instance, while there may be overarching mandates for gender mainstreaming, the absence of specific, actionable policies at the institutional level can result in a lack of accountability and commitment from the faculty and administration. Participants across disciplines reported that the existing CHED-mandated Outcome-Based Education (OBE) curriculum is already packed with technical and content-intensive requirements, leaving limited room to meaningfully integrate GEDI topics. Even when faculty members acknowledge the importance of the GEDI, the pressure to meet technical competencies hinders flexibility. Despite national mandates such as the Magna Carta of Women and CHED Memorandum Orders promoting GAD integration, institutional prioritization remains low. The GEDI is often not seen as essential to academic excellence or accreditation.

*“We already struggle to finish our required topics. Adding new themes like GEDI is difficult unless something is removed,” noted one STEM faculty member:*

*“We get reminded about GAD during Women's Month or when there’s a reporting deadline, but there’s no strategic push from leadership to embed it into our courses,” a program chair shared.*

##### Faculty readiness and resistance

While many faculty members express a need for training in SOGIESC and inclusive pedagogy, others resist personal, ideological, or religious reasons, posing challenges to institution-wide changes. Many faculty members exhibit resistance rooted in misconceptions about the GEDI, such as the belief that it promotes a “*gay agenda*” rather than fostering understanding and respect for diverse identities. This resistance is often compounded by a lack of awareness and training regarding the significance of GEDI themes, which can lead to reluctance to adopt inclusive teaching practices. Furthermore, cultural and religious beliefs may contribute to this resistance, particularly in contexts where traditional values are deeply ingrained. Many faculty members expressed limited knowledge and confidence in addressing gender-related topics. Some feel unequipped to integrate SOGIESC (Sexual Orientation, Gender Identity and Expression, and Sex Characteristics) concepts into content or discussion. Some faculty members also shared that resistance comes not only from a lack of knowledge but also from personal, religious, or cultural beliefs that conflict with inclusive practices. In such cases, discussions on gender diversity are either avoided or dismissed.

*“We haven’t had formal training on gender sensitivity. I’d like to include it in class, but I don’t know how to do it properly,”* said a general education faculty.

*“Some colleagues openly say they don’t want to teach about gender identity because it conflicts with their values,”* one university GAD coordinator disclosed.

##### Marginalization in content and delivery

This theme reveals how exclusionary norms and materials perpetuate invisibility and limit inclusive learning experience. This marginalization is evident in the curriculum, where the narratives and contributions of underrepresented communities, such as LGBTQIA+ individuals, persons with disabilities (PWDs), and indigenous peoples, are frequently overlooked or misrepresented. For instance, portrayals of PWDs in educational materials often lack depth and fail to depict them as capable individuals, which can perpetuate stereotypes and limit their visibility in society. Additionally, the delivery of content may not be sensitive to students’ diverse backgrounds, leading to feelings of exclusion and disconnection from the learning process. This situation is exacerbated by educators’ lack of training on how to effectively integrate GEDI themes into their teaching practices, resulting in a curriculum that does not reflect the rich diversity of student identities. Courses often lack meaningful representation of women, LGBTQIA+ individuals, indigenous people, persons with disabilities, and other marginalized identities. Textbooks and materials mostly reflect dominant cultural narratives. Aside from representation gaps, learning environments often reproduce exclusionary norms through biased language in textbooks, gendered classroom roles, or assumptions in assessment tasks.

*“Most case studies or examples we use feature heteronormative, urban, and able-bodied individuals,”* one faculty member from the Health Sciences revealed.

*“We’re told to use inclusive language, but many of the prescribed materials still use binary pronouns or stereotype women,”* a literature instructor noted.

##### Weak policy implementation

Participants explained that, although policies are formally in place, they are not consistently enforced or clearly translated into curriculum or teaching practices. They noted that while guidelines such as the CHED’s CMO 1 Series of 2015 exist, the absence of clear and actionable directives often results in varied interpretations and inconsistent implementation across institutions. According to them, this vagueness creates a disconnect between the intentions of GEDI policies and actual classroom practices, as many faculty members and administrators are uncertain about how to integrate GEDI principles into their curricula. Participants also emphasized that weak monitoring and evaluation mechanisms further compounded the issue, as there is little accountability to ensure adherence to policies. They pointed out that GEDI initiatives often depend on individual faculty commitments rather than strong institutional mandates, making long-term sustainability difficult. Despite national directives on GAD mainstreaming, participants observed a persistent implementation gap at both institutional and program levels. They reported that policies rarely translate into curriculum plans, teaching guides, or evaluation systems, and even when policies exist, enforcement remains weak, with few accountability structures to support systematic curriculum reviews or consistent faculty compliance.

*“We submit GAD reports, but they are separate from what happens in classrooms,”* a dean commented.

*“It’s easy to claim we follow gender policies, but who checks? Who measures whether we actually do it in courses?”* asked the participants from the Curriculum Development Office.

## Discussion

Based on the first research question, stakeholders perceive the integration of GEDI concepts within the Philippine education curriculum framework as currently characterized by symbolic visibility (vague mentions in documents without concrete outcomes), a recognized need for holistic support systems (mental health, mentorship, inclusive extracurriculars), the necessity of embedding GEDI across core subjects using modular and interdisciplinary contextual approaches, the critical importance of intersectional and locally relevant reforms centering marginalized voices, the requirement to explicitly define and integrate GEDI-aligned competencies, and the essential role of robust monitoring and accountability mechanisms. Studies show that a central concern is the symbolic and inconsistent application of DEI initiatives in the US context, where institutions have mission statements that seem to lack a real impact. For instance, faculty at a U.S. university recognize institutional commitment to DEI values, yet they observe that these values are not sufficiently mirrored in organizational culture or recruitment practices (Beer et al., 2023). Similarly, Moroccan universities face challenges in effectively integrating these principles into their curricula and teaching methods despite ongoing efforts (Abouabdelkader, 2023). The inconsistent integration of discipline-specific practices adds to the complexity, as the adoption of DEI concepts differs significantly across various fields. For instance, computer science faces obstacles such as insufficient faculty support and difficulties in disseminating knowledge to tackle gender disparities (Takeuchi et al., 2024), whereas psychological resources aimed at promoting Equity, Diversity, and Inclusion often neglect to incorporate these principles into their syllabi (Fuentes et al., 2021). Resistance from faculty and a lack of preparedness pose significant challenges, as efforts to integrate inclusive teaching methods show favorable attitudes but highlight an urgent need for additional training and support (Kim et al., 2023). Furthermore, faculty in health professions indicate a necessity for improved knowledge in DEI and cultural competence (Ellis et al., 2023) The ongoing marginalization of underrepresented identities is evident in the challenges related to inclusion stemming from rurality, gender inequity, and epistemic injustice faced by students in Zimbabwe (Simbanegavi and Goronga, 2025). Additionally, diversity initiatives in Belgium often unintentionally perpetuate class, gender, race, and ableist disadvantages with a disproportionate impact on non-white women (Nascimento Rocha et al., 2024). Progress is impeded by inadequate policy enforcement and structural limitations, including the lack of representation of women and people of color in leadership roles, as well as the gender salary disparities noted in McMaster University’s Department of Medicine (Harper et al., 2021).

In research question two, the identified gaps and challenges in integrating GEDI principles within existing curricular structures include uneven implementation across disciplines, non-standardized strategies reliant on individual faculty initiatives rather than institutional mandates, structural constraints, faculty preparedness and resistance, marginalization of underrepresented identities in content and delivery, and weak policy implementation. A study by Raton-Hibanada et al. (2025) in the Philippine setting revealed that the challenges encompass a lack of familiarity with the GEDI, cultural resistance, and resource limitations that impede effective integration. The findings demonstrate notable obstacles to the execution of gender equity and diversity initiatives in academic settings, marked by inconsistent applications across various fields. Research conducted at the University of Genoa highlights the challenges in incorporating Equity, Diversity, and Inclusion (EDI) principles, especially in STEM disciplines (Campanini Vilhena et al., 2024). Additionally, initiatives within the Catalan university system aimed at integrating gender considerations into program evaluations emphasize the ongoing difficulty in achieving consistent implementation across various academic sectors (Verge, 2020). This inconsistency is further exacerbated by the absence of standardized strategies and dependence on individual faculty initiatives. Even national mandates for Gender Equality Plans (GEPs) frequently fall short of effecting meaningful change, primarily due to fragmented adoption and a deficiency in transformative institutional strategies. This situation often leads to reliance on instrumental drivers and normative gender construction (Pinho et al., 2022). Additional challenges encompass organizational limitations and faculty readiness, as institutional barriers and unprofessional behavior obstruct diversity initiatives in academic health centers (Harper et al., 2021). Moreover, structural and cultural elements hinder the inclusion of women in leadership roles in African universities, highlighting the need for systemic reforms (Idahosa, 2021). Resistance from faculty remains a continual challenge, evident in opposition to Gender Action Plans at the project level and reluctance to alter established cultural and behavioral norms that hinder equality initiatives (Pinho et al., 2022).

### Proposed GEDI-responsive curriculum framework in higher education

As the landscape of higher education evolves, it is imperative to create equitable, inclusive, and socially responsive learning environments. In the Philippines, this is both a constitutional mandate and a moral obligation, guided by national and international commitments such as the Magna Carta of Women (RA 9710), the Safe Spaces Act (RA 11313), and the Sustainable Development Goals (SDG 4 and 5). To address these mandates meaningfully within higher education institutions (HEIs), this research proposes a GEDI-Responsive Curriculum Framework—a comprehensive and evidence-based four-layer structure that embeds Gender Equity, Diversity, and Inclusivity (GEDI) into all levels of curriculum design and implementation. The framework is aligned with the higher education curriculum and grounded in intersectional and transformative learning theories.

**Figure 2:**
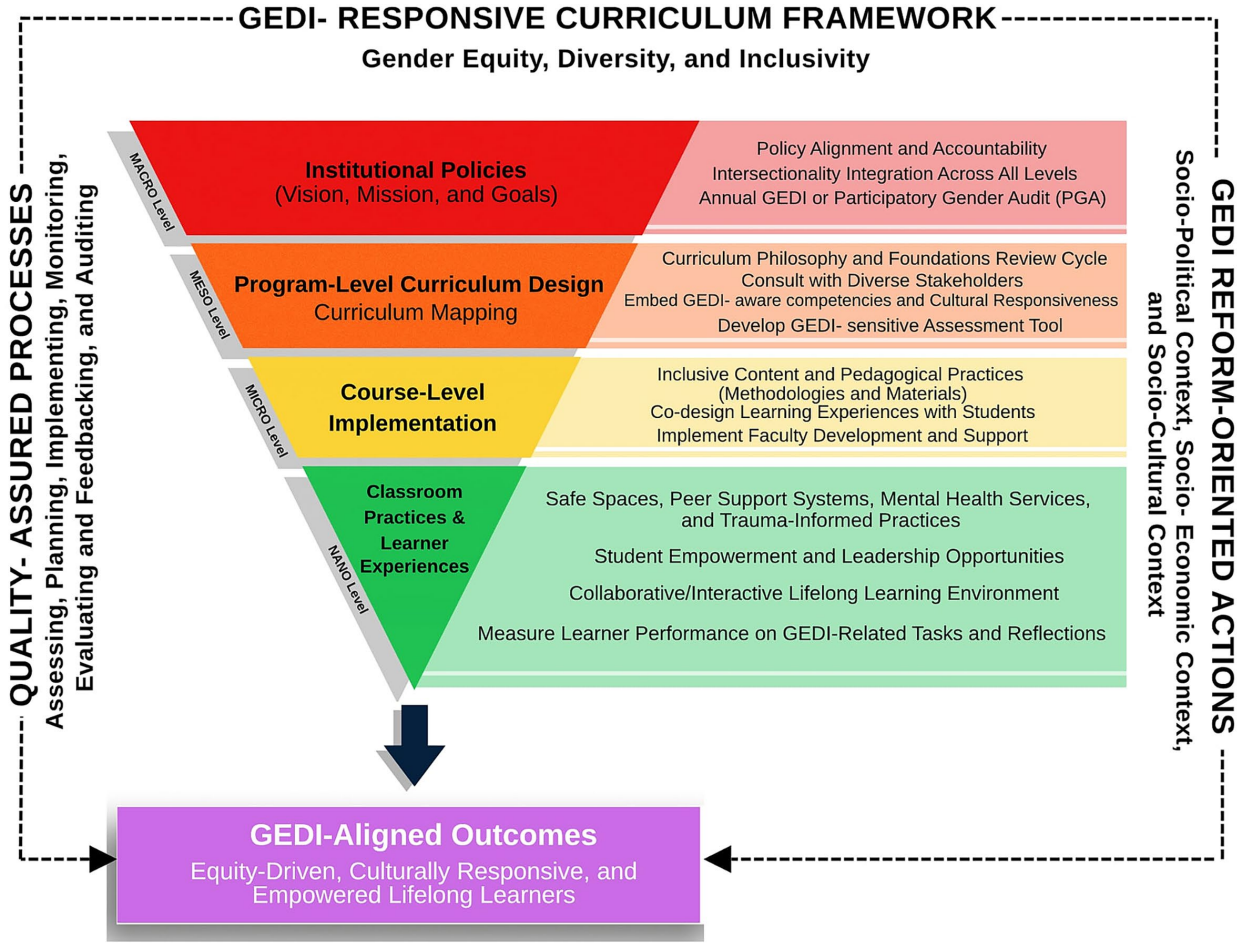
GEDI-responsive curriculum framework.

The purpose of the four-layered GEDI Integration Framework is to offer a structured and context-responsive approach to embedding Gender Equity, Diversity, and Inclusivity (GEDI) across all levels of the education system. At the *macro* level, the framework aims to align national and institutional policies, vision, mission, and goals with legal mandates and global development frameworks to institutionalize the GEDI as a core educational commitment. At the *meso* level, the framework ensures that academic programs intentionally integrate GEDI-aware competencies into Program Learning Outcomes (PLOs), curriculum maps, and instructional designs, fostering socially conscious and inclusive graduates. At the *micro* level, it guides course-level planning and delivery, promoting inclusive pedagogy, contextualized content, and performance-based assessments that reflect GEDI principles. At the *nano* level, it emphasizes the creation of affirming classroom environments and meaningful learner experiences through safe spaces, peer support, and culturally responsive teaching. The overarching purpose is to transform education into a powerful tool for equity and social justice, ensuring that learners are equipped to engage ethically, critically, and compassionately in a diverse, interconnected world (see Figure 2).

### How does intersectionality shape GEDI curriculum demands?

The proposed GEDI-framework (see Figure 2) operates under several foundational assumptions that support its implementation across all the layers. First, it assumes that GEDI is a legal obligation and moral imperative that must be integrated systemically from institutional leadership to everyday classroom interactions. At the *macro level*, it assumes that policies alone are insufficient without accountability mechanisms, and that structural change requires alignment with broader socio-political commitments. At the *meso level*, it presumes that inclusive curriculum design must be intentional and that meaningful change depends on ongoing stakeholder consultation and responsiveness to learners’ diverse realities. At the *micro* level, it assumes that faculty plays a central role in translating GEDI principles into teaching practices, and thus requires sustained capacity-building, reflection, and support. At the *nano* level, the framework assumes that learners are not passive recipients of content but active participants whose voices, identities, and experiences shape the educational process. It also assumes that intersectionality—recognizing overlapping and intersecting forms of oppression—is critical to addressing exclusion. Lastly, transformation is a continuous process that requires regular assessment, feedback, and participatory audits to evaluate progress and adapt strategies over time. These assumptions ensure that GEDI reform is deeply rooted, equity-driven, and responsive to the complex realities of learners and institutions.

### GEDI-responsive curriculum framework implications to policy and practice

1. *Mandate the comprehensive integration of GEDI into all higher education institutions’ curricula via institutionalization and policy directives*. Integrate accrediting frameworks and quality assurance systems with GEDI concepts by embedding pertinent indicators in performance assessments. Establish systematic participatory gender audits (PGAs) to assess compliance and efficacy of the GEDI. Allocate adequate resources to facilitate curricular integration, enhance capacity building, and promote student-centered GEDI programs beyond mere awareness events.
2. *Legal and rights-based curriculum compliance*. Incorporating gender-related national laws into course materials, teaching methodologies, and student participation activities. Incorporate intersectionality into all policy levels by addressing overlapping identities, including sexual orientation, gender identity and expression, and sex characteristics, often pronounced “soh-jee-esque” (SOGIESC), ethnicity, class, and disability.
3. *Facilitate systematic, continuous professional development for faculty on gender sensitivity, inclusive pedagogy, and intersectional teaching methodologies*. Develop inclusive pedagogical activities, contextualized materials, and collaboratively designed learning assignments. Revise assessment instruments to incorporate performance-based, reflective, and equity-centered evaluation methodologies. Establish gender-disaggregated feedback systems to assess classroom inclusion and guide instructional enhancement.
4. *Monitoring and continual improvement.* Create GEDI dashboards and analytics to monitor implementation progress at all levels. Collaborate with external stakeholders (e.g., NGOs and advocacy groups) to produce credible, intersectionality-informed evaluations and policy recommendations.
5. *Promote affirming, courteous, and participatory classroom settings by utilizing inclusive language and standards*. Enable students influence their curriculum and pedagogical choices via feedback mechanisms, focus groups, and leadership positions. Implement support networks, including peer mentorship, culturally attuned mental health services, and inclusive student organizations. Vary assessment formats to cater to different learning styles and facilitate genuine proof of GEDI comprehension.

## Conclusion

This study examines stakeholder perceptions and identifies gaps in integrating Gender Equity, Diversity, and Inclusivity (GEDI) principles into the Philippine higher education curriculum. It proposes a responsive curriculum framework aligned with national mandates and global sustainable development goals. The findings revealed that stakeholders view the integration of GEDI concepts into the higher education curriculum framework as primarily symbolic and inconsistently executed. This approach encounters considerable gaps and challenges, including uneven integration across disciplines, resistance and unpreparedness among faculty, marginalization of underrepresented identities, weak enforcement of policies, structural limitations, and lack of standardized strategies. This study may not provide a comprehensive picture of the Philippines’ diverse educational contexts, as it examined only a limited number of regions. Few faculty members teach higher education programs for Indigenous Peoples (IPs), Persons with Disabilities (PWD), or Special Education (SPED). The results in these areas may not be applicable to other situations; therefore, researchers should exercise caution in generalizing the results. Future research may focus on more comprehensive implementation and the development of systematic feedback systems to assess the feasibility of integrating the GEDI (Gender Equality, Diversity, and Inclusion) framework into higher education institutions. Given that the study included only 19 participants from selected regions, the findings reflect localized perspectives rather than nationally representative trends. Future research involving broader geographic and institutional sampling is needed to verify the applicability of the proposed framework across higher education in the Philippines. Conducting several case studies across various educational settings can yield additional context-specific outcomes and provide a deeper understanding of how the framework can be adapted and sustained in diverse institutional contexts.

## Data Availability

The raw data supporting the conclusions of this article will be made available by the authors, without undue reservation.
